# Alterations to the Kinetic Chain Sequence After a Shoulder Injury in Throwing Athletes

**DOI:** 10.1177/23259671241288889

**Published:** 2024-11-14

**Authors:** Liam P. Owens, Ginny Coyles, Omid Khaiyat

**Affiliations:** † School of Health and Sport Sciences, Liverpool Hope University, Liverpool, UK; Investigation performed at Liverpool Hope University, Liverpool, UK

**Keywords:** shoulder, motion analysis, throwing biomechanics, kinetic chain, segmental motion

## Abstract

**Background:**

Kinetic chain (KC) sequencing is essential for efficient energy translation through the body in overhead-throwing sports. A sequencing breakdown can result in injuries to the throwing shoulder and thus the management of athlete recovery in an attempt to minimize the impact on both training and performance.

**Purpose:**

To determine kinematic differences in KC sequencing, imperative for the prevention and rehabilitation of a shoulder injury, during maximal throwing in overhead athletes with and without a shoulder injury.

**Study Design:**

Controlled laboratory study.

**Methods:**

Kinematic data were collected and analyzed for 36 male overhead athletes with (symptomatic) and without (asymptomatic) a shoulder injury (18 participants per group) during maximal overhead-throwing trials using 3-dimensional motion analysis (100 Hz). Peak angular velocities and associated timing of the throwing shoulder, throwing elbow, thorax, pelvis, lead hip, and rear hip were calculated to determine the KC sequence in both groups. Kinematic data were compared using independent *t* tests, and relationships between variables were assessed using the Pearson correlation coefficient (both *P* < .05).

**Results:**

The KC sequence in overhead athletes with or without a shoulder injury was the same, except for peak elbow extension and shoulder flexion angular velocities. These angular velocities occurred simultaneously in asymptomatic throwers (both 0.17 % before ball release [BR]) but sequentially in symptomatic throwers (0.06 % before BR and 0.67 % after BR, respectively). No differences were evident in stride length (m) or resultant ball velocity (m/s) between the groups, despite differences in key joint angular velocities across KC segments (*P* range, <.001-.035). Relationships between resultant ball velocity and all key joint angular velocities were evident for symptomatic but not asymptomatic throwers (*P* range, <.001-.026).

**Conclusion:**

Our study demonstrated that overhead athletes, regardless of their shoulder injury history, had similar KC sequencing across the lower limb and lumbopelvic-hip complex segments before differences in the timing of peak elbow extension and shoulder flexion angular velocities of the throwing arm approaching BR. Further research investigating muscle activity changes and technique parameters during overhead throwing may present explanations as to how we can ensure that the KC sequence is not altered as a result of an injury.

**Clinical Relevance:**

This study provides a new perspective on the KC and how an injury may not change the sequence itself in overhead-throwing performance.

The kinetic chain (KC) during overhead throwing is an important mechanism for force generation and refers to the transfer of energy from the lower extremity to the core musculature and then to the upper extremity.^12^ The interaction of segments to produce force has since been widely investigated across sporting movements, particularly in overhead sports, to underpin the transfer of energy as a key consideration in achieving the desired outcome. Joint acceleration torques create rapid angular moments of KC segmental masses, which accumulate kinetic energy until it is released,^29^ making the relationships between joint movements essential for ensuring the efficient transfer of energy.

To understand key movements during overhead throwing, phase breakdowns have been proposed across the literature, with 6 phases of the overhead throw outlined^6,9^: windup, stride, arm cocking (AC), arm acceleration (AA), arm deceleration (AD), and follow-through. To consider functionality across the KC during the aforementioned phases of throwing, classifications have been proposed to provide a segmental breakdown of the KC itself. Essentially, 2 main classifications have been described: (1) lower extremity, lumbopelvic-hip complex, upper extremity, and wrist/hand^22^ and (2) lower extremity, pelvis and trunk, scapula, and distal segment.^25^ As initial energy is transferred through the KC from foot contact (FC), it is translated through the lower extremity to the core^20^; up through the trunk, scapula, and upper extremity; and into the throwing object. However, breakdowns in KC sequencing have been reported to result in deficits in the legs, hips, trunk, and scapula and have been identified in 50% to 67% of athletes with shoulder injuries.^15,32^ As such, a decrease of 20% in energy transfer from the trunk to the throwing arm would require an increase of 34% in shoulder internal rotation velocity to achieve the same force output.^14^ This break in KC sequencing requires distal segments to increase their functional capacity to achieve the same force and load within the movement, and this has been described as the “catch-up” phenomenon.^34^ This can result in a mechanical energy transfer rate exceeding the threshold that human tissue is able to withstand, subsequently increasing the probability of shoulder injuries.^23^ Identifying the relationships between segmental joint movements could assist in determining the exact point at which the catch-up phenomenon becomes apparent in overhead athletes, although limited studies have investigated this directly.

Within overhead sports, dominant shoulder injuries are common in 25% to 48% of tennis players^16,26^ and in approximately 23% of English professional cricketers.^27^ Throwing load can be considered a main factor associated with the occurrence of a shoulder injury, with resultant fatigue being a primary factor for KC breakdown. In 1 season, handball players have been reported to have undergone at least 48,000 throwing rotations during both practice and competition.^1^ The large number of rotations increases stress on both the glenohumeral joint and the shoulder girdle and can result in failure.^11^ Shoulder injuries have been reported to change positional capabilities during overhead throwing, particularly during the AC phase. The main impact on mechanics can be seen during external rotation of the throwing arm in overhead athletes who have undergone superior labrum anterior-posterior repair^18^ and athletes with muscle tightness in the latissimus dorsi and subscapularis muscles.^17^ Other mechanical adaptations as a result of an injury are not well published when considering other KC segments.

There are limited studies that have directly investigated the KC sequence during overhead throwing. The angular velocity order in male javelin throwers was found to be lower trunk forward tilt, lower trunk left tilt, upper trunk left rotation, right shoulder internal rotation, abduction, horizontal adduction and right elbow extension together, and finally, right wrist flexion.^19^ In handball players, movements of both the pelvis and trunk are key to momentum generation and throwing velocity in addition to the relationship between internal rotation of the throwing shoulder and ball velocity.^33^ The importance of the KC is evident across the literature, particularly in exercise prescription for rehabilitation after an injury. However, integrating these approaches into everyday training to target lower and upper extremity impairments through synergistic movement patterns,^35^ specifically the synchronicity of throwing shoulder and hip movements^13^ as well as the rotations of the pelvis and trunk. While segmental links and proximal-to-distal sequencing mechanics have been described, consideration of the differences in KC sequencing and the association between proximal and distal segmental movements in overhead athletes with and without a history of shoulder injuries have not been investigated. Therefore, the purpose of this study was to investigate KC sequencing during overhead throwing in athletes with and without a shoulder injury. It was hypothesized that (1) differences would be evident when comparing the KC sequence of asymptomatic and symptomatic throwing athletes and (2) higher joint angular velocities would be apparent in the upper limb for symptomatic athletes.

## Methods

A total of 36 male participants were recruited from local, regional, and university sport clubs involving a predominant overhead-throwing skill (ie, baseball, cricket, handball) and were required to have played regularly within the past 3 years. The participants were classified into 2 groups: asymptomatic (n = 18; mean age, 25.1 ± 6.7 years; mean height, 182.0 ± 5.0 cm; mean weight, 87.4 ± 22.4 kg) and symptomatic (n = 18; mean age, 32.1 ± 10.7 years; mean height, 182.3 ± 8.1 cm; mean weight, 87.6 ± 16.5 kg). Inclusion criteria were based on a history of shoulder injuries, with asymptomatic defined as those who had never had a history of injuries to their throwing shoulder or upper limb. Symptomatic participants had a clinical history of shoulder injuries (ie, shoulder instability or rotator cuff–related disease) within the last 3 years as well as difficulty or pain during performance, as indicated in the sports module of the Quick Disabilities of the Arm, Shoulder and Hand questionnaire. As a result of their shoulder injury, all symptomatic participants had been prescribed rehabilitation exercises alongside pain management (ie, prescribed medications and/or injections) after consulting medical professionals, with 2 throwers undergoing a surgical intervention. All symptomatic throwers had returned to team training/competition in their sport before participating in this study. All participants were tested during a 1-day collection session between July 2017 and March 2019. Previous literature has reported differing KC sequences between male and female throwers,^19^ and as a result, female throwers were omitted from this study. The research project received ethical approval from a National Health Service Research Ethics Committee (REC reference: 16/NW/0049; IRAS ID: 183797).

There were 35 spherical reflective markers (diameter: 9.5 mm) attached to anatomic landmarks in accordance with the Plug-in Gait full-body model (Nexus; Vicon Motion Systems). Also, 3 more spherical markers (diameter: 19 mm) were added to the participant to aid pelvic and thorax tracking: 2 attached to the lateral edges of the iliac crest and 1 to the left upper back. The upper arm marker on the throwing arm was attached to an elastic wristband positioned over the biceps and pointed outward; this was to reduce marker movements during overhead-throwing trials. Wrist markers were attached to a small wand threaded through elastic wristbands in accordance with the Plug-in Gait model. The 4 head markers were attached to an elastic headband after it was positioned on the participant’s head. For ball tracking purposes, 4 reflective markers (diameter: 9.5 mm) were attached to a tennis ball.

Participants performed a brief warm-up that reflected their normal preparation before sport participation. Before any overhead-throwing measurements, participants familiarized themselves with the throwing technique.^6^ The windup phase, including the knee-up position, was omitted to allow the normalization of the throwing action across participants from different overhead sports. Participants performed 5 successful maximal-effort overhead throws at a target (dimension: 1200 × 800 mm) using a tennis ball (diameter: 67.1 mm; weight: 56.8 g). A throw was deemed successful if the ball struck any part of the target area, positioned 3.5 m away.

Overall, 8 Vantage cameras (Vicon Motion Systems), sampling at 100 Hz and with a camera threshold of 0.2, were used to collect kinematic data during overhead-throwing movements. There were 2 high-speed digital video cameras (1 MP; sampling at 100 Hz; Bonita 720c [Vicon Motion Systems]) synchronized in Nexus to provide a visual representation of movements to assist with phase identification (ie, ball release [BR]). In addition, a 600-mm × 400-mm × 100-mm piezoelectric force platform (Type 9281EA; Kistler) was used in combination with Nexus (sampling at 1000 Hz) to aid the detection of FC during throwing trials.

Upon trial completion, the reflective markers were tracked and filtered (Woltring filter at 10 Hz), with kinematic and marker trajectory data exported for analysis. Key temporal phase markers were identified using a combination of angle data outputs, force platform data, and Bonita camera recordings. The total throw time for each trial was calculated as the time between start (lead leg toe-off) and end (rear leg toe contact) of the overhead throw. Other key phase markers (FC, maximal external rotation [MER], BR, and maximal internal rotation [MIR]) were determined from time after start (ms) and converted to a percentage of the throw duration. Differences in the percentage between phase markers denoted the duration of each throwing phase. Key joint angular velocities for lead hip, rear hip, pelvis, thorax, throwing shoulder, and throwing elbow were calculated for each throwing trial and the time-to-peak values calculated relative to BR (%). Resultant ball velocity was calculated as the distance traveled by the ball from BR to 0.1 seconds after. Stride length (m) was calculated using a combination of lead foot toe and heel marker trajectories within Nexus, with overlaid Bonita camera footage. Stride length was determined from the point at which the lead foot toe left the ground and the point at which contact of the foot was re-established. Confirmation of FC at the end of the stride phase was attained from the ground-reaction force measured by the piezoelectric force platform.

Statistical analyses were undertaken using SPSS software (Version 25; IBM). The Shapiro-Wilk test was conducted to confirm or reject the normal distribution of each dataset, with the level of significance set at *P* < .05.^8^ Independent *t* tests were performed to assess statistical differences between the groups for peak joint angular velocities (deg/s), timing of peak joint angular velocities (% throw relative to BR), stride length (m), phase duration (%), and resultant ball velocity (m/s). The *P* value was set at <.05 for all variables. To assess relationships between variables (key angular velocities and ball velocity), the Pearson correlation coefficient was used, with the *r* value reported alongside the *P* value, with the latter set at *P* < .05.

## Results

Throwing phase duration was only greater for the AC phase (*t*[175] = 3.373; *P* = .001), with asymptomatic throwers spending longer in that phase (14.3% ± 4.4%) than symptomatic throwers (12.0% ± 4.3%). No other phase duration differences between groups were evident for the stride (asymptomatic: 37.3% ± 8.6%; symptomatic: 37.4% ± 9.3%), AA (asymptomatic: 3.2% ± 1.4%; symptomatic: 3.4% ± 1.8%), AD (asymptomatic: 19.5% ± 5.0%; symptomatic: 20.4% ± 5.3%), and follow-through (asymptomatic: 25.8% ± 10.1%; symptomatic: 26.8% ± 10.8%) phases. No differences were recorded between the groups for stride length (asymptomatic: 0.95 ± 0.26 m; symptomatic: 0.90 ± 0.28 m) (*t*[175] = 1.230; *P* = .220). In addition, no differences in stride length presented as a percentage of the thrower’s height were evident (asymptomatic: 51.9%; symptomatic: 49.3%) (*t*[175] = 1.185; *P* = .237).

**Figure 1.**
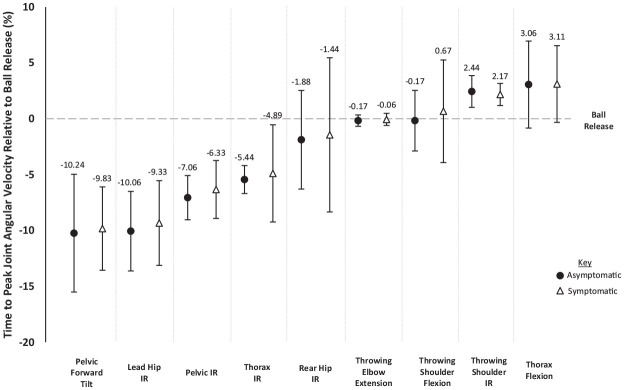
Timing of peak joint angular velocities (mean ± SD) relative to ball release (%) in asymptomatic and symptomatic throwers. IR, internal rotation.

No differences in the time-to-peak values of key joint angular velocities (%) relative to BR could be identified between the 2 groups (Figure 1). The order of angular velocities forming the KC sequence was the same between groups, except for elbow extension and shoulder flexion angular velocities occurring simultaneously in the asymptomatic group (both 0.17% before BR) but consecutively in the symptomatic group (0.06% before BR and 0.67% after BR, respectively). In contrast, the peak joint angular velocities recorded were greater for the asymptomatic group for lead hip internal rotation (*t*[113.704] = 4.155; *P* < .001), rear hip internal rotation (*t*[170] = 2.745; *P* = .007), pelvic rotation toward the target (*t*[168.542] = 2.619; *P* = .010), and thorax rotation toward the target (*t*[175] = 2.302; *P* = .023) (Table 1). Symptomatic throwers exhibited greater pelvic forward tilt angular velocity (*t*[164.310] = −2.127; *P* = .035).

**Table 1:** Peak Joint Angular Velocities and Associated Throwing Phases for Asymptomatic and Symptomatic Throwers^*a*^

	Pelvic Forward Tilt	Lead Hip IR	Pelvis IR	Thorax IR	Rear Hip IR	Elbow Extension	Shoulder Flexion	Shoulder IR	Thorax Flexion
Asymptomatic									
Angular velocity, deg/s	124 ± 49^*b*^	224 ± 148^*b*^	432 ± 96^*b*^	646 ± 105^*b*^	166 ± 89^*b*^	1727 ± 360	627 ± 239	4050 ± 1430	129.9 ± 56.0
Phase	AC				AA			AD	
Symptomatic									
Angular velocity, deg/s	142 ± 61^*b*^	151 ± 64	391 ± 108	607 ± 120	131 ± 77	1634 ± 333	615 ± 262	4147 ± 1515	149.6 ± 81.0
Phase	AC				AA			AD	

a Data are shown as mean ± SD. Negative values indicate before ball release, and positive values indicate after ball release. AA, arm acceleration; AC, arm cocking; AD, arm deceleration; IR, internal rotation.

b *P* < .05.

Relationships were identified between several key joint angular velocities across the KC. Most consecutive peak joint angular velocities in the identified KC sequencing order (Figure 1) showed significant relationships. For the asymptomatic group, significant relationships between joint angular velocities were identified between pelvic forward tilt and lead hip internal rotation (*r* = 0.242; *P* = .026), lead hip internal rotation and pelvic rotation toward the target (*r* = 0.312; *P* = .004), pelvic rotation toward the target and thorax rotation toward the target (*r* = 0.521; *P* < .001), throwing elbow extension and rear hip internal rotation (*r* = 0.346; *P* = .001), throwing elbow extension and throwing shoulder flexion (*r* = −0.484; *P* < .001), and throwing shoulder flexion and throwing shoulder internal rotation (*r* = 0.556; *P* < .001); no relationships were evident between thorax rotation toward the target and rear hip internal rotation angular velocities, or between thorax flexion and throwing shoulder internal rotation angular velocities. For the symptomatic group, significant relationships between joint angular velocities were identified between pelvic forward tilt and lead hip internal rotation (*r* = 0.253; *P* = .018), lead hip internal rotation and pelvic rotation toward the target (*r* = 0.279; *P* = .009), pelvic rotation toward the target and thorax rotation toward the target (*r* = 0.688; *P* < .001), thorax rotation toward the target and rear hip internal rotation (*r* = 0.437; *P* < .001), throwing elbow extension and throwing shoulder flexion (*r* = −0.698; *P* < .001), and throwing shoulder flexion and throwing shoulder internal rotation (*r* = 0.711; *P* < .001); no relationships were evident between throwing elbow extension and rear hip internal rotation angular velocities, or between thorax flexion and throwing shoulder internal rotation angular velocities.

No differences between groups were recorded for resultant ball velocity (asymptomatic: 21.6 ± 3.0 m/s; symptomatic: 21.4 ± 3.4 m/s) (*t*[175] = 0.343; *P* = .732). However, all joint angular velocities were found to have significant relationships with resultant ball velocity (*P* < .05) for symptomatic throwers. This was similar in asymptomatic throwers, except for rear hip internal rotation (*P* = .812) and thorax flexion (*P* = .194).

## Discussion

The main objective of the present study was to determine differences in KC sequencing during maximal throwing in overhead athletes with and without a shoulder injury. The KC sequence between asymptomatic and symptomatic throwers was similar across the lower limb and lumbopelvic-hip complex during the early phases of the overhead throw. Timing differences for peak elbow extension and shoulder flexion angular velocities were evident approaching BR, occurring simultaneously in asymptomatic throwers (both 0.17 % before BR) but sequentially in symptomatic throwers (0.06 % before BR and 0.67 % after BR, respectively). In addition, differences in peak angular velocities of key joints through the KC were evident, with greater peak values for lead and rear hip internal rotation (*P* < .001 and *P* = .007, respectively) as well as pelvic and thorax rotation toward the target (*P* = .010 and *P* = .023, respectively) in asymptomatic throwers. Symptomatic throwers only exhibited greater peak angular velocity for pelvic forward tilt (*P* = .035). These findings may provide reasons for why KC differences were evident between the groups approaching BR.

The initial stride of overhead throwers is an important stage of KC sequencing, and its impact on kinematics of the throwing action has been widely reported.^4,7,30^ During the stride phase, potential energy is developed and converted into kinetic energy, and it is then transferred through the lower limb segment and up the KC.^20^ The appropriately established initial base of support allows subsequent sequential rotation of KC segments to transfer energy through the body to the ball.^4^ The present study identified no differences in stride length between the groups, with stride length presented as a percentage of the thrower’s height substantially lower for both groups compared to the 76% to 85% values reported in previous literature.^4,7^ Differences reported may be a result of sport-specific movements, with previous literature focusing on baseball pitching, which included the knee-up phase marker^6^ and subsequently increasing forward momentum and a greater stride length. Hence, it can be proposed that there was no difference between the throwing groups in initial kinetic energy conversion before FC and KC sequencing processes.

The KC sequence in both groups was identified from peak angular velocities and followed a similar progression across lower limb and lumbopelvic-hip complex segments. A previous angular velocity order has been reported in javelin throwers,^19^ and the findings of the present study agree with those findings until the involvement of the throwing arm in the KC sequence. However, the present study also considered other key joint angular velocities to determine differences across the KC during overhead throwing, specifically as a result of shoulder injury history. To date, there is limited information available describing changes in KC sequencing during maximal overhead throwing in athletes with and without a shoulder injury. Asymptomatic throwers were found to exhibit significantly higher peak hip (both lead and rear), pelvic, and thorax rotational angular velocities. In contrast, symptomatic throwers exhibited higher peak pelvic forward tilt angular velocity during the AC phase and throwing shoulder internal rotation angular velocity during the AD phase. This suggests that symptomatic throwers had to increase throwing shoulder internal rotation angular velocity and subsequent thorax flexion angular velocity to compensate for lower generated rotational angular velocities of proximal segments and the energy transfer from the lower limb to the throwing arm to attain a similar ball velocity after BR. This would be in line with previously suggested compensation strategies as a result of decreased energy transfer through the trunk.^14^ Interestingly, no differences between the groups were identified for maximal angular velocities that occurred toward the end of the KC sequence, namely, throwing shoulder internal rotation, throwing shoulder flexion, elbow extension, and thorax flexion angular velocities. In line with the “summation of speed” principle,^2^ peak rotational angular velocities in consecutive segments were achieved for both throwing groups, although it was evident that rotation of the thorax (distal) was already initiated before maximal rotational angular velocity was achieved in the proximal segment (pelvis).

Key movements of the pelvis and thorax occur during the AC phase, resulting in peak angular velocities being achieved as the body is rotated toward the target before BR.^30,33^ Symptomatic throwers achieved greater peak pelvic forward tilt angular velocity during the early stages of the AC phase, suggesting that this group may have lost stability of the lead leg after FC. KC energy transfer after lead leg contact is translated through the lower limb to the core.^20^ Therefore, it can be postulated that a loss of lower limb stability after FC resulted in greater pelvic forward tilt angular velocity and may have impeded energy transfer up through the KC. This potential loss of energy transfer through the lumbopelvic-hip complex would require increased angular velocities of the throwing shoulder to compensate and achieve a similar force output. This is in line with previously proposed shoulder angular velocity increases being required^14^ and the importance of pelvic/trunk movements approaching BR.^21^ As a result, increased loading of the throwing shoulder can increase the likelihood of an injury and may have been a contributing factor to injury occurrence for throwers in the symptomatic group. Subsequent peak rotational angular velocities of the lead hip, pelvis, and thorax were reached in the mid-to-late AC phase, with the asymptomatic group achieving greater values for each. Pelvic rotational angular velocities of both groups were comparable to previously published peak angular velocities of between 400 and 700 deg/s,^10,20,31^ albeit at the lower end of the range.

The angular velocity values recorded for throwers within the present study may vary from those previously reported in the literature because of changes in the throwing protocol employed (ie, removal of the knee-up component as outlined by Escamilla and Andrews^6^) and could be a result of the decreased stride length recorded by both groups. While we acknowledge some differences between the sports of the participants within this study, the intention to standardize the throwing action was to ensure that the KC was the main factor for investigation. In addition, the standardized throwing protocol does reflect commonly used throwing techniques in outfielders in cricket and baseball as well as a shooting variation in handball. However, it is possible that the reduced stride length recorded in both throwing groups may have impacted the lead hip angle at FC and resulted in a restriction of pelvic movement ranges^3^ and subsequent reduced angular velocities. Similar differences in angular velocity of the thorax rotating toward the target were evident between groups; the recorded values are much less than previously reported values of 848 deg/s^28^ and 1227 deg/s.^21^ Regardless of potential stride length restrictions, the relationship between the pelvis and thorax for KC sequencing should not be underestimated. A stretch-shortening cycle in the core musculature plays a substantial role in building tension and elastic energy through changes in the degrees of separation between the 2 segments^5^ as they initially rotate away from the target and then toward the target approaching BR. However, the relationships identified between peak pelvic and thorax rotational (toward the target) angular velocities were significant in both groups. This suggests that despite differences in peak angular velocities, the breakdown in the KC did not occur during this stage of the movement.

As the thorax rotates toward the target, the throwing shoulder flexes, adducts, and internally rotates, with the elbow extending approaching BR.^9,10,30^ Within the KC, differences in the timing of elbow extension and shoulder flexion angular velocities were identified, occurring simultaneously in the asymptomatic group and consecutively in the symptomatic group. Interestingly, for the symptomatic group, peak shoulder flexion angular velocity was achieved after BR and just before peak shoulder internal rotation angular velocity. These findings suggest that the reduced energy transfer as a result of lower peak thorax and pelvis rotational angular velocities could have been compensated for by shoulder movements in the symptomatic group. To attain the same resultant ball velocity as the asymptomatic group, additional force would need to have been generated to achieve the same outcome as that of asymptomatic throwers to complete the KC.

The recorded angular velocities for the shoulder and for thorax flexion suggest that symptomatic throwers employed a catch-up strategy similar to that proposed by van der Hoeven and Kibler^34^ in which additional force and stress were placed on the shoulder and increased sagittal-plane mechanics for both the shoulder and the thorax were necessary to generate increased forward momentum. Peak shoulder internal rotation angular velocities of both groups are comparable to the findings of Roach and Lieberman^28^ (4290 deg/s) but less than those reported in professional throwers (7724 deg/s).^21^ It should be noted that the difference here may also be a result of anthropometric variations between the populations examined, as segmental rotation velocities will be impacted by the length of the segment. However, to further support the theory that symptomatic throwers used compensatory mechanisms, both rear hip internal rotation and thorax flexion angular velocities were found to have significant positive relationships with resultant ball velocity. Such relationships were not evident for asymptomatic throwers. This suggests that symptomatic throwers were more reliant on their rear hip angular velocity to aid rotation toward the target; it is possible that this could be a result of poor positioning of the lead leg at the end of the stride phase and subsequently significantly lower pelvic rotation toward the target compared to asymptomatic throwers. In addition, although there were no differences in thorax flexion angular velocity between the groups, it appears that symptomatic throwers were reliant on this to generate resultant ball velocity compared to asymptomatic throwers. This may help to account for the significantly higher rotational angular velocities recorded for asymptomatic throwers earlier during KC sequencing, with symptomatic throwers were more reliant on sagittal-plane movements of the pelvis (start of the KC) and thorax (end of the KC) to generate the same resultant ball velocity.

### Limitations

There were some limitations to this study. Throwing athletes were recruited from different sports (baseball, cricket, handball), and the throwing techniques were normalized using an established throwing protocol.^6^ We deemed this to be comparable to the throwing action used in the outfield in both cricket and baseball and on court in handball. In addition, the main focus was on identifying differences in KC sequencing between asymptomatic and symptomatic throwers; thus, establishing a normalized technique was essential. However, this may have resulted in differences to previously published values for peak angular velocity because of the lack of sport-specific application. All athletes in this study also completed maximal overhead throws using a tennis ball, which is smaller and lighter than balls used in other overhead sports. It has previously been reported that any increase in the weight of the ball can result in 2 potential mechanical adaptations to throwing performance, namely, alterations in the position of the throwing shoulder and leading with the elbow during the AA phase.^24^ Therefore, the smaller and lighter ball used in this study could have resulted in slight alterations to the throwing mechanics and subsequently reduced the load placed on the throwing shoulder in both groups. Additionally, the Plug-in Gait full-body model used for data collection may have underestimated the angular velocities that the shoulder joint achieved. The method of defining the shoulder joint with Vicon software may not have provided a true representation of shoulder movements because we did not account for the complexity of numerous degrees of freedom modeling across the 4 main joints. However, we were satisfied with the methods aiding clear conclusions regarding the main stated objective. Finally, we noted differences in the age of the throwing groups, with older athletes in the symptomatic group. It is considered that this is a result of longer periods of practicing and competing in their sport and having undergone more excessive rotations, increasing their prevalence for shoulder injuries over time.

## Conclusion

The findings of this study indicated a difference in KC sequencing between asymptomatic and symptomatic throwers, with peak shoulder flexion and elbow extension angular velocities occurring simultaneously in the asymptomatic group but sequentially in the symptomatic group. Although no differences were identified for stride length or resultant ball velocity at the start and end of KC, respectively, lower pelvic and thorax rotational angular velocities in symptomatic throwers resulted in greater angular velocities of the throwing arm. This suggests increased loading and stress of the shoulder joint to achieve the same endpoint as asymptomatic throwers and supports findings of previous studies investigating throwing biomechanics. Coaches and rehabilitators should increase focus on movements of both the pelvis and thorax to ensure that energy transfer through the KC is as efficient as possible. This study provides a new perspective on the KC and how a shoulder injury may not change the sequence itself in overhead-throwing performance.
